# The Possibilities of Using Chromium Salts as an Agent Supporting Treatment of Polycystic Ovary Syndrome

**DOI:** 10.1007/s12011-019-1654-5

**Published:** 2019-02-04

**Authors:** Anna Piotrowska, Wanda Pilch, Olga Czerwińska-Ledwig, Roxana Zuziak, Agata Siwek, Małgorzata Wolak, Gabriel Nowak

**Affiliations:** 1 Department of Biochemistry and Basics of Cosmetology, University of Physical Education, Kraków, Poland; 2 Department of Pharmacobiology, Jagiellonian University Medical College, Kraków, Poland; 3 Department of Neurobiology, Laboratory of Trace Elements Neurobiology, Institute of Pharmacology PAS, Kraków, Poland

**Keywords:** Chromium picolinate, Obesity, Insulin sensitivity, Mood disorders, Dietary nutrient, Physiological benefits

## Abstract

The polycystic ovary syndrome (PCOS) is the most frequent endocrinopathy in women in reproductive age with the so far undetermined causes of development. In the etiopathogenesis of PCOS, the role of insulin resistance is emphasised, which was an indication for the attempts at using chromium III salts (Cr) in augmenting pharmacotherapy applied in patients. The analysis of the usefulness and efficacy of this approach was the direct goal of this thesis. Animal tests confirmed the efficacy of chromium in maintaining the appropriate level of glycaemia and insulinaemia, normalisation of plasma concentrations of microelements and also a correlation between the Cr level, insulin and dehydroepiandrosterone (DHEA) was found. A decrease in the expression of 3β-hydroxysteroid dehydrogenase and 17β-hydroxysteroid dehydrogenase was identified in adipose tissue. Clinical studies, although sparse, show that the supplementation with chromium can improve BMI and the parameters evaluating the control of glycaemia and increase the chances for ovulation and regular menstruation. However, the small number and a variability in study protocols makes comparing them very difficult. A completely new subject that has not been yet studied is the possibility of using chromium in levelling mood disorders in patients with PCOS. Currently, there are still no sufficient proofs for introducing chromium as a standard in treating and preventing insulin resistance in patients with PCOS. However, this direction remains open, and treating insulin resistance is an important challenge in clinical practice.

## Introduction

Polycystic ovary syndrome (PCOS) is one of the most frequent endocrinopathies in women in reproductive age and in adolescence. In accordance with American data, this disorder is diagnosed in 4–12% of women; however, according to European data, this condition may occur twice as frequent [1–3]. The frequency of diagnosing in a given country is influenced by the prevalence of obesity and also ethnic and racial differences, as described in the epidemiological literature and in the clinical image of this disorder [4]. The causes of PCOS are still being studied; there is an ongoing search for the genes responsible for the manifestation of the disease, and the clinical image is extraordinarily heterogeneous [2, 5]. There are gynaecological and metabolic symptoms; all of them influence patient’s psychological condition and self-esteem [6, 7].

One of the most important elements of the image of the observed metabolic disorders is insulin resistance and a whole set of glycaemic disorders. In patients with PCOS, obesity and dyslipidaemia are more frequent [8, 9]. This led to the hypothesis that the application of supplementation with chromium (III) indicated as an microelement facilitating in maintaining normal glycaemia and normal level of lipoprotein in plasma and dealing with obesity [10].

## Review Method

A review of Internet databases (PubMed, Ebsco and ScienceDirect) and medical journals was performed, searching for papers in Polish and in English from years 2000–2018. The following keywords and their combinations were used: polycystic ovary syndrome (PCOS, *zespół policystycznych jajników*), *chromium* (*chrom*), *chromium picolinate* (*pikolinian chromu*), chromium chloride (*chlorek chromu*), glucose intolerance (*nietolerancja glukozy*), lean body mass (*beztłuszczowa masa ciała*), lipid profile (*profil lipidowy*), mood disorders (*zaburzenia nastroju*) and depression (*depresja*). The analysis included original, case studies and review papers related to the studied subject.

## Results

### Polycystic Ovary Syndrome

PCOS was described for the first time by Stein and Leventhal in 1935. The first name of the condition was based on both their names. The current terminology is derived from the characteristic image of an ovary with peripherally located cysts. PCOS is characterised by hyperandrogenism and ovary function and changes in their structure [2, 11]. Other names of the syndrome include functional ovary androgenism, chronic hyperandrogenic lack of ovulation, ovary dysmetabolic syndrome, sclerotic ovary syndrome, polycystic ovaries or the aforementioned historical name: Stein–Leventhal syndrome.

### PCOS Symptoms and Course of the Disease

Currently, in order to diagnose PCOS, the Rotterdam criteria were approved by the American Society for Reproductive Medicine and the European Society of Human Reproduction and Embryology, in accordance with which PCOS is indicated by two of the three symptoms: menstruation disorders or amennorrhea with ovulation disorder or lack thereof; clinical and/or biochemical signs of hyperandrogenism; presence of polycystic ovaries in ultrasound, after prior elimination of other conditions related to hyperandrogenism [12].

PCOS frequently presents with acne, hirsutism, hair loss, irregular menstruation and infertility. It increases the risk of endometriosis and breast cancer, and it is also related to the following disorders: dyslipidaemia, hypertension, cardiovascular diseases and diabetes, fitting the consequences of a metabolic syndrome [13]. Hyperandrogenism manifests by androgenic alopecia, hirsutism, acne lesions, increased hair loss, oily skin, seborrhoeic lesions and clitoromegaly. Hirsutism, one of the most frequent symptoms, assessed according to the Ferriman–Gallwey score, is the presence of gruff, thick and pigment-saturated hair in women in places typical for men, e.g. upper lip, chin, chest, nape of the neck, lumbar region, abdomen, thighs and feet [14]. A frequently appearing skin symptom in women with PCOS is also acanthosis nigricans, usually located on elbows, nape of the neck, armpits and under breasts [15, 16]. More than half of the women suffering from PCOS experience overweight and obesity, usually of the androidal type [8]. Increased body weight has negative influence on carbohydrate economy, which causes insulin resistance, leading also to hyperinsulinaemia. It is thought that high concentration of circulating insulin contributes to the increased production of androgen in an ovary and to ovulation disorders. In a significant part of patients, an abnormal lipid profile is observed. Patients with PCOS are at high risk of developing cardiovascular disease being its distant consequence [17, 18].

PCOS is a condition strongly affecting mental health and social functioning. In women with PCOS, an increased number of anxiety and/or depression incidents is reported [6, 7]. In great majority of cases, this is connected with the clinical image of this disorder, deteriorating the quality of life [6, 19]. It has been concluded that the increased level of free testosterone in the plasma, one of the main anomalies in PCOS, is correlated with the increased risk of depression. There are studies reporting even sevenfold increase in the frequency of suicides among patients [20]. Patients often have a problem with their identity and social functioning. They feel the difference between their appearance and the image of the woman created by media. They withdraw from social life. Also, the infertility present in 72% of patients contributes to the development of serious psychosocial problems [8, 19, 21].

### Dietary Treatment and Supplementation in PCOS

In the aspect of direct correlation between obesity and the frequency of PCOS, it seems that diet therapy should play the major role in managing the condition [22]. Studies revealed disorders in the metabolism of many nutrients, such as vitamin D, numerous minerals and omega-6 fatty acid [23]. Therefore, dietary strategies and supplementation have become points of interest of persons studying the problems of women with PCOS [24]. Among others, the effects of dietary supplementation with the application of inositol and folic acid have been indicated [25, 26], and also interesting effects have been observed regarding 12-week probiotic supplementation [27].

Minimising insulin resistance can play a significant role in controlling PCOS [28]. The development and understanding of the phenomenon of insulin resistance in the pathogenesis of this syndrome were an indication to search for new methods, including adjusting the diet of patients [29]. One of the methods leading to this effect was supposed to be chromium (III).

### Chromium

Chromium (Cr) can be present at different levels of oxidation; however, only Cr (III) is responsible for beneficial effects [10]. The first publication on the biochemical role of Cr in mammal organisms was published in 1959 [30], and since that time, it has been placed on the list of the elements necessary for optimal functioning of living organisms. This was the result of observations indicating that the amount of Cr in the structures of hair, plasma and sweat has negative correlation with age, which differentiates it from most contaminations accumulated in organisms with the passing of time. In patients subjected to long-term parenteral nutrition, symptoms of Cr deficiency were observed, manifested mainly by glucose intolerance. These changes were easily treated after supplementation [31, 32]. Indications for its supplementation used to be very broad; however, currently, Cr is one of the elements causing the greatest controversies. It is argued that it does not belong to the necessary microelements, but it evokes a potential benefit and/or adverse effects. Adequate intake (AI) was set based on estimated mean intakes and amounts 35 μg/day and 25 μg/day for young men and women, respectively. In Poland, there are no separate recommendations for this biometal AI, and no detailed guidelines on doses of PCOS supplementation have been set.

### Chromium, Glycaemic Level and Insulin Sensitivity

The first studies of the influence of Cr on maintaining the proper glucose tolerance in rats were conducted in the late 1950s [30]. These observations were confirmed also in clinical setting, in patients subjected to long-term parenteral nutrition [33]. Chromium has become a popular subject of studies regarding glucose intolerance and insulin resistance. Currently, the conclusions are that only supplements including yeast enriched with this metal have influence on the decrease of concentration of glucose or glycated haemoglobin in blood [34]. However, in the paper of Yin et al. comparing the studies from years 2000–2012, it was shown that supplementation can cause decrease of fasting glycaemia, but it has no influence on the concentration of glycated haemoglobin [35]. It is indicated that Cr takes part in insulin signalling and increases sensitivity to insulin of insulin-sensitive cells [36–38], and deficiency, rare as it is, increases the risk of metabolic diseases and diabetes [38].

### Chromium and Lipid Metabolism

Animal study results confirm the positive influence of Cr on lipid profile change [39] and the influence of supplementation with this microelement on full cholesterol concentrations, LDL and HDL cholesterol and triglycerides [40]. However, clinical studies provide ambiguous results. Despite positive observations from the turn of the twentieth and twenty-first centuries [41], the studies of Kleefstra et al. [42] did not achieve positive results of a 6-month supplementation with chromium picolinate (III) (500 and 1000 μg/day). Only a slight dependence between the content of Cr (III) in the serum and the improvement of lipid indicators in the blood was shown. The lack of influence of supplementation is also confirmed by the later studies with the use of yeast enriched with Cr [34] and chromium picolinate (CrP) [43].

### Chromium and Body Mass Control

Chromium dietary supplements were very popular in the 1990s and later, as products facilitating weight loss and increasing muscle mass. To this day, many of them are still offered by pharmacies and drugstores, which, in the context of the frequency of obesity in women with PCOS, is another indication at the necessity of analysing the activity and efficacy of such supplementation. Unfortunately, so far there have not been any scientific reports proving the efficacy of supplementation with Cr in decreasing the content of adipose tissue and increasing muscle mass, against the marketing claims of the manufacturers of dietary supplements [10]. A paper summarising the results of publications regarding the application of supplementation with Cr in persons with overweight and obesity indicates that there have been a relatively small number of studies with proper protocols: high heterogeneity of intervention types and sample sizes, which usually were small or moderate (622 subjects evaluated in total) [44]. The intervention time was 12–16 weeks, and the observation for potential adverse effects: 8–24 weeks. The results show that Cr supplements in all doses have some influence on losing weight after 12–16 weeks of supplementation, but the effective dosage cannot be yet determined. No decisive proofs in the form of significant changes of BMI, waist circumference or percentage content of adipose tissue were found. This is significant in the light of the studies conducted by Pazderska et al., which indicate that waist circumference in patients with PCOS is suggested as a predictor of glucose and lipid metabolism disorders [45]. In the review of 10 studies performed by Pitter [46], a significant differential effect to the benefit of CrP was indicated; however, the clinical significance of this effect is still disputed. Also, the safety of supplementation with CrP in persons with overweight and obesity is still being determined, although reports on adverse effects of supplementation with Cr are more casuistic in nature [47].

### Supplementation with Chromium in PCOS

Due to the concomitance of PCOS and insulin resistance, an attempt was made to apply supplementation with Cr in this disorder. Studies conducted in animals showed the efficacy of the increased supply of Cr in maintaining the proper level of insulin and glucose in fasting conditions, an improvement of the microelement levels in plasma, which were disturbed due to a disease [48], and they confirmed the dependence between the concentration of Cr, insulin and dehydroepiandrosterone (DHEA) [48, 49]. Thanks to the mouse model of PCOS, the influence of the syndrome on phenotypical characteristics was demonstrated: the mice developed abdominal obesity, hyperandrogenism, hyperinsulinaemia, follicular atresia and light hepatic steatosis. The model mice with Cr supplementation showed a tendency to decrease body weight gain, liver mass and its steatosis was significantly lower in these animals; however, Cr did not improve the morphology of ovaries. In the adipose tissue of the supplemented animals, a decrease of 3β-hydroxysteroid and 17β-hydroxysteroid dehydrogenases was revealed. In model animals, a reverse correlation between the concentration of Cr in serum and in muscles and the concentration of insulin was noted, which points to the fact that PCOS can cause a low level of Cr in serum and in muscles, and it seems to lead to hyperinsulinaemia [47]. The analysis of plasma concentrations of selected metals, including Cr, was the subject of several papers and was reviewed in detail in 2016 [50]. Two studies evaluating plasma chromium levels selected by Spritzer et al. did not indicate differences between women with PCOS and controls.

As of now, several randomised placebo-controlled clinical studies have been conducted in patients with PCOS. Amr and Abdel-Rahim evaluated the influence of supplementation in adolescents (age range, 14–17 years old) [51]. Thirty-five adolescent girls with histories of menstruation disorders were qualified for the study. In 6 months, subjects were given 1000 μg of CrP. After this period, the lack of significant change of BMI was indicated; however, the number of subjects with oligomenorrhoea/amenorrhoea decreased in the supplemented group, and also a significant decrease of the mean ovary volume and the total number of ovarian cysts was observed. What is important, in the supplemented group, the level of free testosterone in the serum was significantly lower. However, no significant improvement was noted in the image of acne and hirsutism.

The rest of the studies of the application of therapy or supporting therapy of PCOS with Cr were conducted in mature women, and the meta-analysis of the results is presented in review studies [52–55]. In Heshmati’s work [53], the final meta-analysis included only five properly randomised and placebo (or an approved drug)-controlled studies, with 137 women with PCOS in total. The authors indicated three studies assessing Cr as a single supplement [56–58]. Two of them assessed Cr in comparison to metformin [59, 60]. Populations of the studied groups were from 6 to 46 women, and the applied diagnostics of PCOS complied with the criteria of the Rotterdam consensus [12]. These studies were conducted in the USA, Egypt and Iran. All the women were in reproductive age, and the intervention time was from 6 weeks to 6 months. Chromium, in comparison to placebo or other therapies, caused significant changes in the insulin resistance markers (summary data analysis from five studies indicated significantly lower HOMA-IR values in the group supplemented with chromium). The study describing the influence of supplementation on HOMA-B showed a significant difference. In this study, after 8 weeks of supplementation in women with PCOS, also a significant decrease in HOMA-IR and an increase of the QUICKI result were reported, in comparison to placebo [57]. Comparing Cr with metformin treatment, it was indicated that supplementation of diet with Cr was not related to insulin concentration [59, 60]. Meta-analysis conducted by Tang et al. [55] indicates the improvement in insulin sensitivity; however, it has failed to show a beneficial effect of Cr supplementation on the level of total and free testosterone. Data analysis showed no effect of supplementation on body mass, fasting insulin and glucose or serum lipid levels. Also, there were no significant differences in luteinizing hormone (LH), follicle-stimulating hormone (FSH) and prolactin (PRL). Maleki concludes that a longer period of supplementation is required to obtain a significant effect of Cr intake on the level of sex hormones in the plasma [54]. The conducted studies and their meta-analyses do not provide an unequivocal answer regarding the efficacy of supplementation, and the observed effects are too poor to allow for the implementation of such an approach to the standard therapy of patients with PCOS.

### Chromium and Treatment of Mood Disorders in PCOS

According to Kurek and Babiarczyk, due to the multidimensional character of the problems related to PCOS in women, they should be provided complex care including the physical and psychosocial problems [6]. Treatment of physical disorders should be conducted in accordance with the current needs, which is aimed at reaching the goals they have chosen. The psychosocial disorders, which are very often skipped in the process of treatment, should be paid special attention, and these women should be provided psychological help in fighting depression, which often appears in this condition [6, 7]. The first information indicating the possibility of antidepressant activity of Cr is from the 1990s. It was described that the symptoms of atypical depression, dysthymia, seasonal affective disorders and daily mood swings decreased. Due to the fact that in some women with PCOS depressive states related to unsuccessful attempts at getting pregnant, high BMI and low self-esteem, preparations with Cr can have positive influence; however, this has not been studied so far.

The mechanism of antidepressant activity of Cr seems to be of pharmacological nature [61–63], as deficiency of this metal is rare [64, 65], and the effective doses are relatively high both in animal tests [61–63] and in clinical application: Cr salt, administered in dose 400–600 μg/day, was effective in patient with depression [66–68].

The antidepressant activity of Cr studied in animal models of depression depends (to various degrees) on noradrenergic, dopaminergic and serotonin signalling. It was shown that the antidepressant activity of chromium chloride (CrCl_3_) is partially dependent on serotonin receptors 5-HT_1A_ and 5-HT_2A_ [63]. On the other hand, the noradrenergic mechanism was indicated in the studies with the application of adrenergic receptor antagonists (propranolol, prazosin, yohimbine) and the reinforcement of the antidepressant activity of reboxetine (selective inhibitor of noradrenaline uptake) [63]. Also, the role of dopaminergic system was indicated [63]. The involvement of the monoamine systems was also indicated by Franklin and Odontiadis [69], who demonstrated increase in the level of serotonin and its metabolites, as well as a change in the number of 5HT_2A_ receptors of the downregulation type. Glutamatergic system takes part in the antidepressant activity of Cr through receptors AMPA (α-amino-3-hydroxy-5-methyl-4-isoxazolepropionic acid receptor) and NMDA (N-methyl-D-aspartate receptor) [62]. Additionally, Khanam and Pillai [70] suggested the involvement of K^+^ channels; however, this has never been confirmed. The complex mechanism in correlation with an interesting clinical profile (activity in dysthymic, seasonal disorders and atypical depression) and the influence on carbohydrate metabolism, and perhaps also lipid metabolism, makes Cr supplementation in patient with PCOS a still interesting study subject.

## Summary

Chromium gained its greatest popularity by the end of the twentieth century. In scientific literature, one can find an enormous number of studies concerning the influence of supplementation on carbohydrate–lipid economy of the body, but not only on that aspect. The indications regarding the influence on the markers describing insulin resistance allowed to presume the efficacy of such supplementation in PCOS.

The indicated mechanisms of action for Cr confirmed in animal tests are unfortunately not always confirmed in the conducted clinical studies. The efficacy of supplementation in diabetes and dyslipidaemias is undermined, in maintaining appropriate body weight—negated. The available reports on the role of Cr are often contradictory, and the state of knowledge does not confirm the previous assumptions fully, which, unfortunately, often is the result of poor planning of research works and especially using very small patient groups.

The role of Cr supplementation in PCOS remains unsolved. An interesting phenomenon is especially the possibility of this element influencing the mood, which constitutes a big challenge in the discussed condition. A significant factor for using Cr supplementation in patients with PCOS can be also the fact that in animal tests, low Cr level was observed in serum and in muscles with concomitant hyperinsulinaemia. Nevertheless, in order to unequivocally assess the efficacy of Cr supplementation in the discussed condition, it is necessary to conduct further studies in this direction of sufficient sample size for sufficient duration in well-defined populations.
